# Recognizing the Degree of Human Attention Using EEG Signals from Mobile Sensors

**DOI:** 10.3390/s130810273

**Published:** 2013-08-09

**Authors:** Ning-Han Liu, Cheng-Yu Chiang, Hsuan-Chin Chu

**Affiliations:** Department of Management Information Systems, National Pingtung University of Science & Technology, 1, Shuefu Road, Neipu, Pingtung 912, Taiwan; E-Mails: djmb3567@gmail.com; (C.-Y.C.); sassnd@msn.com (H.-C.C.)

**Keywords:** electroencephalogram, attention status, electroencephalography (EEG) classification, support vector machine

## Abstract

During the learning process, whether students remain attentive throughout instruction generally influences their learning efficacy. If teachers can instantly identify whether students are attentive they can be suitably reminded to remain focused, thereby improving their learning effects. Traditional teaching methods generally require that teachers observe students' expressions to determine whether they are attentively learning. However, this method is often inaccurate and increases the burden on teachers. With the development of electroencephalography (EEG) detection tools, mobile brainwave sensors have become mature and affordable equipment. Therefore, in this study, whether students are attentive or inattentive during instruction is determined by observing their EEG signals. Because distinguishing between attentiveness and inattentiveness is challenging, two scenarios were developed for this study to measure the subjects' EEG signals when attentive and inattentive. After collecting EEG data using mobile sensors, various common features were extracted from the raw data. A support vector machine (SVM) classifier was used to calculate and analyze these features to identify the combination of features that best indicates whether students are attentive. Based on the experiment results, the method proposed in this study provides a classification accuracy of up to 76.82%. The study results can be used as a reference for learning system designs in the future.

## 1. Introduction

Whether students are attentive when learning significantly influences their learning outcomes. In traditional face-to-face instruction, teachers generally observe students' expressions to determine whether they are sufficiently attentive. However, this method is excessively subjective and consumes a significant amount of the teacher's energy. Furthermore, besides face-to-face instruction, students may engage in distance learning over the Internet, which further increases the difficulty of determining whether students are attentive. In all circumstances, the neurons in the human brain are ceaselessly active, emitting small amounts of electromagnetic waves. These electromagnetic waves are used as electroencephalography (EEG) signals. Without training, humans are generally unable to control fluctuations in their EEG signals. Therefore, the use of EEG signals to determine whether students are learning attentively is viable. Based on the frequency range, EEG signals can be divided into the following five wavebands [1]:

- α activity: electromagnetic waves ranging between 8 and 13 Hz in frequency, and between 30 and 50 μV in amplitude. This type of periodic wave is produced in the parietal and occipital regions of the brain when in a state of consciousness, quiet, or at rest. When thinking, blinking, or otherwise stimulated, α waves disappear. This is known as an alpha block.
- β activity: electromagnetic waves ranging between 14 and 30 Hz in frequency, and between 5 and 20 μV in amplitude. This type of activity occurs in the frontal region when people are conscious and alert. These waves are particularly apparent when a person is thinking or receiving sensory stimulation.
- θ activity: electromagnetic waves ranging between 4 and 7 Hz in frequency, with an amplitude of less than 30 μV. This activity primarily occurs in the parietal and temporal regions of the brain. Such waves are produced when people experience emotional pressure, interruptions of consciousness, or deep physical relaxation.
- δ activity: electromagnetic waves ranging between 0.5 and 3 Hz in frequency, and between 100 and 200 μV in amplitude. In a conscious state, most adults exhibit almost no δ activity; instead, this activity occurs when in a deep sleep, unconscious, anesthetized, or lacking oxygen.
- γ activity: electromagnetic waves ranging between 31 and 50 Hz in frequency, and between 5 and 10 μV in amplitude. Recent studies have found that γ activity is related to selective attention. Other studies have also highlighted that this activity is related to cognition and perceptual activity.

Because various regions of the brain produce EEG signals, cerebral electromagnetic activity is traditionally collected using the international 10–20 electrode placement system (10–20 System), which involves attaching electrodes to 37 locations on the skull. Although this method facilitates the observation of all EEG signal changes, practically applying this technique to students is extremely inconvenient and impractical. Because a person's emotions, mental state, and attentiveness are governed by various parts of the brain in the forehead region, observing the EEG signals from this area is a viable method for determining whether students are attentive. To facilitate the wearing of electrodes, this study adopted a commercialized mobile wireless EEG signal detection device for detecting and analyzing EEG signals of the frontal lobe. Because few previous studies have employed classrooms as the research environment, this study uses EEG signals as the medium to observe students' attentiveness during learning. The characteristics of EEG signals during states of attentiveness were identified by applying classifiers to the observed EEG data. The objective of this study was to successfully observe and identify whether students are attentive using simple EEG signal detection and classification. By employing the proposed system, teachers and the students themselves can assess their attentiveness during instruction, enabling both parties to implement necessary adjustments. Thus, the basic process of determining whether students are attentive involves classifying EEG signals. This study adopted a support vector machine (SVM) that produces excellent two-class results as the classifier. Mobile devices were employed to collect learners' EEG signals, which were then used to calculate various features. The effect that these features had on the classification performance was then analyzed. The remainder of this paper is organized as follows: Section 2 discusses relevant research; Section 3 describes the extracted features and the EEG classifier; Section 4 outlines the experimental results, and Section 5 presents the conclusions.

## 2. Related Research

Beebe *et al.* investigated whether sleep disorders during puberty cause inattention and consequently affect learning. The results indicated greater θ activity when the subjects were inattentive [2]. Vidal *et al.* suggested the concept of brain-computer interface (BCI) technology [3,4], primarily regarding using BCI systems as communication tools between humans and the world, transforming detected EEG signals into commands, and realizing actions through hardware equipment. As published in *Neuroscience Letters*, Scherer *et al.* developed a system [5] for classifying EEG signals using Fisher's linear discriminant analysis (FLDA) and a virtual keyboard for spelling. This involved constructing a system based on the EEG signals produced when a person thought of the three directions down, left, and right. In 1977, researchers analyzed the power spectrum of EEG signals and found that it can reflect fluctuations in the level of alertness [6]. Scholars have also used EEG signals to evaluate mental exhaustion [7,8], identify feelings after listening to music [9] or observing various sports [10], assess emotions [11], control the movement of small mechanized vehicles, and alter the height of hospital beds.

Research regarding EEG signals, concentration, and learning remains immature because scholars and researchers have only recently begun exploring this field. A further motivation for such research is to facilitate long-distance learning [12,13]. Because actual face-to-face contact between teachers and students is impossible with long-distance learning, the difficulty of assessing students' learning state is greater than that with the face-to-face teaching method. Therefore, scholars and researchers have begun examining the relationship between EEG signals, learning, and concentration. In 2001, Gerlic *et al.* [14] observed alpha activity to examine the differences in EEG signals generated by beginner teachers compared to professional teachers, and by teaching courses that employ textual materials compared to multimedia materials. The results showed that learning ability is better controlled under the instruction of professional teachers, and multimedia material more easily stimulates brain activity compared to textual material. Yaomanee *et al.* [15] identified locations on the scalp that are suitable for detecting attention related EEG signals. The study involved three experiments (*i.e.*, reading a book, locating 3D figures, and answering questionnaires) for determining whether the subjects were attentive. A two-minute piece of music was played to all subjects before the experiment to encourage relaxation, and any EEG signal changes during this period were observed. The results showed that α activity was slightly higher when the subjects were in a relaxed state, whereas β activity was greater when the subjects were attentive. Li *et al.* [13] developed an emotional learning system that was subsequently combined with the two classification methods of *k*-nearest neighbor (*k*NN) and naive Bayes. Students' emotions and attitudes were analyzed according to the alpha and theta activity-related EEG signal electrical potential collected from 13 locations using the previously mentioned classification methods to identify their level of attentiveness. The experiment results showed a maximum identification rate of 66.7%; however, the average rate was 44.4%. Li *et al.* [16] conducted EEG examinations using brain power-related tasks and asked the subjects to report their level of attentiveness. They employed *k*NN classification as the research method, and designed a system for instantly measuring people's level of attentiveness. The classification accuracy of the system was 57.3%. Belle *et al.* [17] employed EEG and electrocardiography (ECG) to compare their classification accuracy for attentiveness. The experiments involved the participants watching an interesting and tedious video clip for 20 min each before performing classification calculations using regression, C4.5, and random forest. The results of these methods for examining biological data show that the classification accuracy of EEG is 8.74% higher than that of ECG. This finding indicates that EEG is an appropriate technique for observation. In addition, EEG involves a substantial amount of data and is worthy of continued research and additional applications.

SVM is a supervised learning algorithm for solving binary classification problems that was proposed by Vapnik in 1995 [18]. SVM is generally applied to statistical classification and regression problems [19]. Cuingnet *et al.* [20] combined SVM and the Laplacian classification method to increase the ease of resolving cerebral image rendering. This technique can also be employed to render 3D images and images of the area between the two layers of the cerebral cortex. However, a comparison and classification of cerebral images obtained from 30 elderly subjects and 30 Alzheimer's patients indicated that cross-section cerebral images are identical to the images rendered using the technique described previously. Costantini *et al.* [21] applied SVM classification to EEG signals to design a BCI system capable of developing simple mathematical calculations or identifying the signals that indicate movement of the left or right hand. Jrad *et al.* [22] proposed using a weighted SVM to design a framework for classifying EEG signals. The results showed that a sensor weighting SVM (sw-SVM) can effectively classify datasets and error-related potential (ErrP) datasets. This method is also suitable for classifying the event-related potential (ERP) of small-scale training samples.

## 3. Feature Extraction and EEG Classification

Although every person's attentiveness to the same learning content differs, and their EEG signal fluctuations vary, this study aims to identify the changes in EEG signals during attentive learning under normal conditions by using convenient and simple methods. In a controlled learning environment, the influence (attentive or inattentive) that the experiment had on the students and EEG-related data was determined and analyzed. An SVM classifier was then employed to identify the classification information, thereby achieving the study objective.

Brainwave sensors were adopted to collect the subjects' EEG signals when learning. EEG sensing and data processing modules were then used to filter and prepare the collected data. The features of EEG signals after processing were separated into the two categories of attentive and inattentive, and employed as the SVM classifier training set. After optimizing the SVM classifier configurations, the classifier was used to instantly detect and identify the participants EEG data during instruction to assist teachers with assessing the students' attentiveness levels.

### 3.1. EEG Sensors

**Figure 1.**
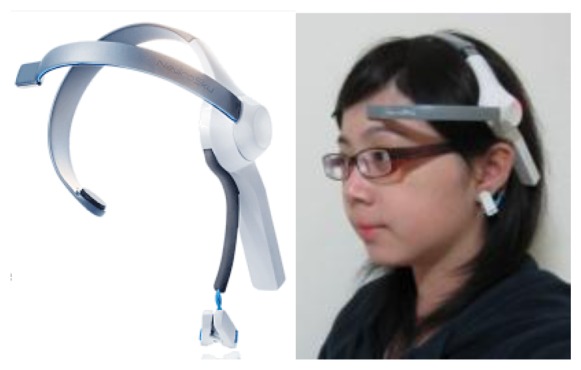
The brainwave sensor and application method.

To facilitate within-classroom use by students, this study employed highly portable mobile brainwave sensors, as shown in Figure 1. These brainwave sensors detected and digitized weak EEG signals produced by the brain and then wirelessly transmitted them to the hardware equipment. The EEG signal detection device employed for this study was MindSet, which features a single dry sensor. Ground and reference sensors were placed on the subjects' left ear. Wireless Bluetooth connectivity facilitated mobility. Sample rates as high as 512 Hz delivered raw signals in the alpha, beta, delta, gamma, and theta bands. The brainwave sensors use ThinkGear AM chip module technology to identify and digitize weak EEG signals, and can filter and extract electrical signals, such as the electrical waves produced by muscle movement, from the surrounding environment. The module chips collect, filter, augment, perform A/D conversions, process, and analyze EEG signals, before transmitting the processed and digitized EEG signals to the hardware equipment.

**Figure 2.**
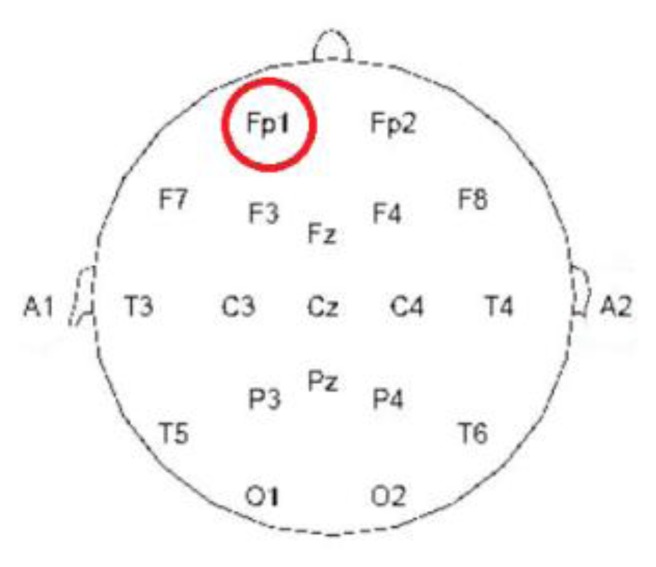
International 10–20 electrode placement system.

After a century of experimentation, experts in the field of neuroscience have defined the various areas that control corporal movement in the cerebrum. For example, the top of the cerebrum controls the limbs, and the posterior of the cerebrum controls sight. Because all areas of the cerebrum can produce EEG signals, electromagnetic waves are traditionally collected according to the international 10–20 electrode placement system (10–20 System), which involves placing 37 electrodes on the skull. Although this method facilitates observation of EEG signal changes, applying this technique to students is inconvenient and impractical. Because human emotions, mental states, and levels of attentiveness are controlled by the cerebral cortex in the forehead, detecting the EEG signals produced in this area of the brain is a viable method for determining whether students are inattentive. In this study, the brainwave sensors collected EEG signals from Fp1, as shown in Figure 2. The sensor manufacturers placed the sensor at Fp1 because of the similarity between Fp1 and Fp2 signals and because this placement is similar to the mechanical design typically used for headphones. Generally, dry sensors are prone to environmental influences (e.g., motion artifacts); thus, errors exist in the detected signals. Although the sensor in the proposed identification system is a dry sensor, it is easy to wear and economic and, thus, can be extensively employed for teaching. Therefore, this study examined the feasibility of differentiating between attentiveness and inattentiveness based on EEG signals acquired using the proposed sensor.

### 3.2. Feature Extraction

EEG data were recorded at a sampling rate of 512 Hz with a 16-bit quantization level. To simplify the data processing method, EEG data were processed using a low-pass filter with a cut-off frequency of 50 Hz. A fast Fourier transform (FFT) was employed to transform a segment with 512 sampling points to the frequency domain. The segment was slid with 256 points overlapping the previous segment. Assuming *F*(*n*) (*n* = 1, 2, …, 512) is the FFT result of a segment, the associated Power Spectral Density (PSD) is as follows:

(1) $$P(n)=\frac{F(n)F*(n)}{N}$$

where F*(*n*) is the conjugate function of *F*(*n*) and *N* = 512.

Five types of brainwaves exist in EEG signals, namely, α, β, δ, θ, and γ. However, research [23] has identified that the waves most related to human mental states are α, β, δ, and θ. The energy value is summed according to the waveband distribution of the EEG signals to produce four features. Assuming that *P_freq_* is the energy value of frequency *freq*, the features can be defined as follows:

(2) $$E_{\alpha }=\sum_{\text{freq}=8}^{13}P_{\text{freq}}$$

(3) $$E_{\beta }=\sum_{\text{freq}=14}^{30}P_{\text{freq}}$$

(4) $$E_{\theta }=\sum_{\text{freq}=4}^{7}P_{\text{freq}}$$

(5) $$E_{\delta }=\sum_{\text{freq}=0.5}^{3}P_{\text{freq}}$$

In addition, according to previous research [23], definite interrelations exist between α and β activities. For example, α activity indicates that the brain is in a state of relaxation, whereas β activity is related to stimulation. In the study mentioned previously, to observe continuous changes in the mental state of the subjects, the ratio of α and β activities was used as the feature for assessing the level of mental attentiveness. This study produced the following feature value using the same principle:

(6) $$R=\frac{E_{\alpha }}{E_{\beta }}$$

where *R* is also a feature for determining whether students are attentive. Therefore, in this study, five features are extracted as the basis for classification. In this study, the EEG signal data of the test subjects was manually separated into attentive and inattentive, and used to determine the recognition accuracy rate after completing calculations and classifications using the classifier.

### 3.3. SVM Classifier

**Figure 3.**
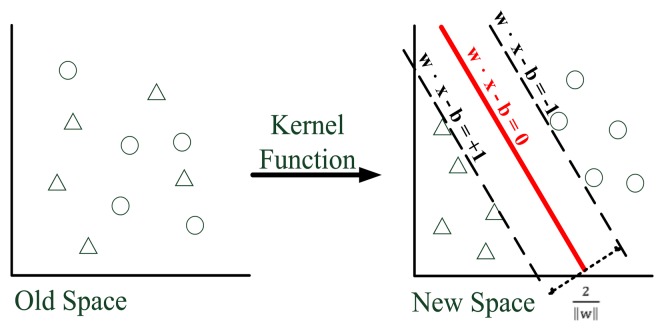
Example SVM.

This study employed SVM classifiers with an excellent classification algorithm to separate EEG signals into the two categories of attentive and inattentive. Ideally, an SVM can identify a hyperplane that separates attentive and inattentive EEG signal data in its high-dimensional feature spaces. The basic concept of an SVM is as follows: Each sample in a set of training samples is matched to two categories before being reflected into a high-dimensional space using the Kernel function [24]. Subsequently, the SVM attempts to create a model and uses it to assign the samples to a category. The model then constructs a separating hyperplane in the high-dimensional space (denoted using the solid red line in Figure 3, Old Space). On either side of the hyperplane, which divides the samples, parallel hyperplanes are located (denoted using dotted black lines in Figure 3, New Space). The SVM maximizes the distance between these two parallel hyperplanes. A greater distance or difference between parallel hyperplanes indicates a smaller total SVM error rate.

According to the pretest results, polynomial kernel functions offer superior classification accuracy. Therefore, this study used a polynomial kernel function to project feature vectors into high-dimensional space before performing SVM calculations and classification. Furthermore, *k*-fold cross-validation was used to verify the recognition accuracy of the SVM classifier. Collected EEG signal data were randomly partitioned into non-repeating *k* subsample sets. One set was retained as validation data, and *k*-1 sets were used as training data. This process was repeated *k* times to determine the average value, which denoted the recognition accuracy rate.

## 4. Experiments

**Figure 4.**
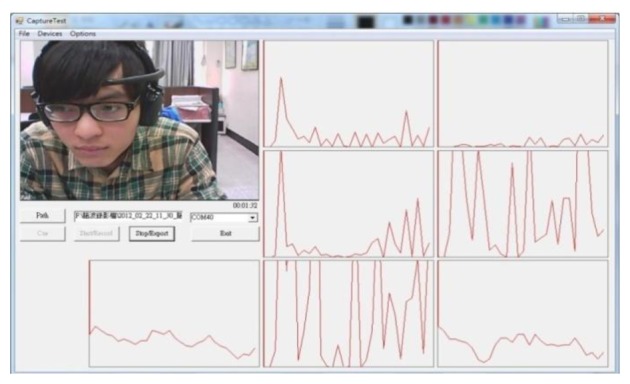
Real-time brainwave recording system.

**Figure 5.**
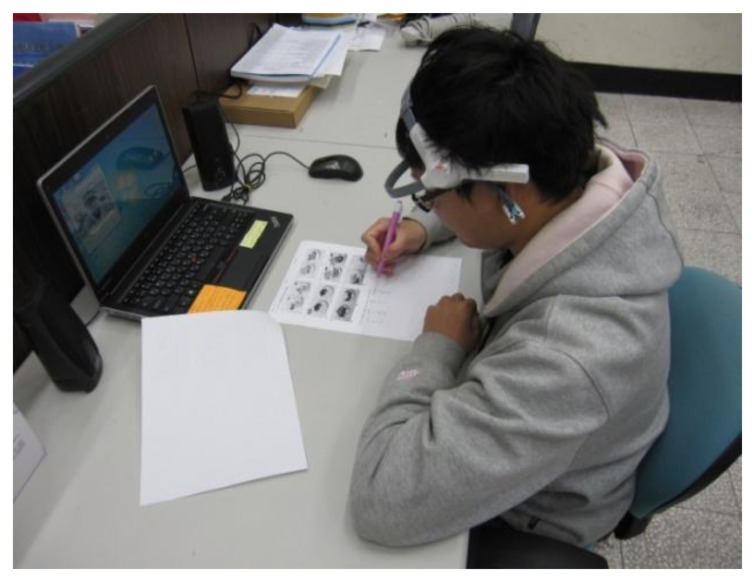
Experimental environment.

Currently, an approved EEG signal database regarding attentiveness and inattentiveness is not available for analysis. The production of attentive and inattentive EEG signals when learning is an extremely difficult and complex process. This study designed a controlled environment and guided the subjects in producing the required EEG signal data. Based on the preliminary determination results and the subjects' subjective perceptions, these data were labeled according to category. Signals that were non-definitive were considered invalid. To accurately record the EEG signals of the test subjects and identify their level of attentiveness, in addition to recording their EEG signal data during the experiment, their facial image and the surrounding sounds at the time of the experiment were also recorded to facilitate effective differentiation in post-experimental data analysis. The brainwave recording system is shown in Figure 4, and the experimental environment is shown in Figure 5.

### 4.1. Experiment Setting

This study recruited 24 test subjects (12 men and 12 women), with an average age of 25 years (the subjects' age ranged between 22 and 27 years). The hearing ability, mental state, and health conditions of the test subjects were normal. Furthermore, the test subjects had not undergone any EEG-related training. Since several studies maintain that the brainwave performance of males and females differs, this study aimed to design a gender dependant identification system. In the other words, two classifiers were developed for the proposed system and the EEG signals for male and female subjects were processed individually. Moreover, this study endeavored to design a system that does not require advanced collection of the users' EEG signal data. Thus, two classifiers (*i.e.*, one for male and one for female) capable of determining users' attentiveness and inattentiveness after training were developed.

To clearly identify the state of attentive EEG signals when students are learning, standard English class material was used as the experiment material for this study. The experiment involved the test subjects listening to English phrases and then answering related questions (question types: ordering the images, selecting answers based on illustrations, and multiple choice), to ensure that the test subjects could concentrate during the experiment. When the experiment was initiated, the test subjects were asked to wear the EEG sensor for 2 min to familiarize themselves with the sensor to prevent potential discomfort during use from influencing the accuracy of the experiment results. The volume of the speakers was also tested to ensure that the test subjects could conduct the experiment at the most appropriate volume. Each test subject was required to complete the English test under two scenarios. The first scenario was without interference, where the test subjects listened to English conversations before answering questions. The second scenario included the distraction of two people conversing while the test subjects listened to English conversations. They then answered questions and reported the content of the overheard conversation. The scenarios were designed to induce inattentive behavior when the test subjects completed the English examination. During the data collection experiment, 4291 unprocessed entries of EEG row data were collected. The length of each datum was 1 s. The test subjects' conditions were manually determined. For the first and second scenario, the subjects were predetermined to be in an attentive and inattentive state, respectively. All collected data were examined, and the researchers and subjects reviewed video footage of the experiment together to determine whether the subject was in an attentive or inattentive state. If the subject was unsure of their mental state during the experiment, the particular datum was discarded. For example, in the first scenario, if the subject was unsure of whether they were attentive, the corresponding EEG signal was excluded. For the second scenario, if the subject was hesitant regarding whether they were inattentive, the corresponding brainwave was rejected. Once the test subjects' EEG data and data regarding their attentiveness were obtained, the feature values of attentive and inattentive EEG signals were marked. After eliminating ambiguous data, 4,289 and 2,674 attentive and inattentive EEG signal samples were obtained. To prevent the number of attentive and inattentive samples from affecting the classification accuracy of the classifier, 2,400 samples (1,200 samples from the male subjects and the others from the female subjects) each were randomly selected from the attentive and inattentive samples. Consequently, energy conversion was conducted on the original EEG signals to produce characteristics for classification.

For the SVM classifier, this study used two types of polynomial kernel functions and adjusted the cost parameter to analyze which combination of features can be used to obtain the optimum classification results. The polynomial kernel functions were as follows:

Polykernel:

(7) $$K\left(x_{i},x_{j}\right)={\left(x_{i}^{T}\cdot x_{j}+1\right)}^{d}$$

Normalized polykernel:

(8) $$K\left(x_{i},x_{j}\right)={\left(x_{i}^{T}\cdot x_{j}+1\right)}^{d}/\sqrt{x_{i}^{T+1}+x_{j}^{T+1}}$$

### 4.2. Experimental Results

This study originally employed a single feature for classification; however, the accuracy rate did not exceed 47%. Therefore, feature combinations were used as a basis for the classification system. First, the degree of influence that each feature has on the classification accuracy was determined. The analysis results are listed in Table 1, which shows the average classification accuracy and the highest classification accuracy. To the classifiers for male and for female, the k value of k-fold was set as 5, which means 240 samples were used as testing samples.

Table 1 shows that the polykernel classification accuracy rates all slightly exceed those of the normalized polykernel. Moreover, the results in Table 1 show that SVM can provide greater accuracy when all features are employed.

This study analyzed the accuracy rate for attentive and inattentive EEG signals when all features are used. If the classification accuracy rate of attentiveness is known as the attention rate (AR), the total number of attentive entries is called total attention (TA), and the correct classification of EEG signal data into the attentive category is labeled correct for attention (CA):

(9) $$AR=\left(\frac{CA}{TA}\times 100\right)\%$$

Assuming that the classification accuracy rate of inattentiveness is called the inattention rate (IR), the total number of inattentive entries is known as the total inattention (TI), and the correct classification of EEG data into the inattentive category is labeled correct for inattention (CI).

(10) $$IR=\left(\frac{CI}{TI}\times 100\right)\%$$

The number of TAs and TIs included in the sample was 2400 each. Table 2 shows the AR and IR of post-classification EEG data when the polykernel kernel function is employed. The data in Table 2 show that a greater cost parameter denotes a lower AR and a higher IR.

### 4.3. Discussion

Table 1 indicates that the experiment data obtained in this study are unsuitable for normalized polykernel. Another inference based on Table 1 is that all five features have a significant influence on classification because removal of any feature causes a slight decline in both the highest and average classification accuracy rate compared to when all features are used. This indicates that each feature has a positive influence on the classification accuracy rate. However, according to the result of ANOVA (analysis of variance) testing, the contributions of features are different. Specifically, features δ and R exert the greatest influence on the classification accuracy. About the δ feature, the experiment result matches the conclusion in the literature [25] which stated that changes in delta activity are related to linguistic acquisition. Because the learning method in our experiment belongs to linguistic instruction, the δ feature value is an important basis for classification. Although previous studies have contended that increased θ activity and reduced β activity respectively related to attention and inattention show minimal contributions from other wavebands, the experiment results of this study show that all features influence classification to some extent. The possible reason is that the limitations of a dry sensor for the detection location Fp1, only by using all wavebands can superior classification results be obtained.

Table 2 shows that the ratio of correct attentive classification entries (AR) was higher than that of correct inattentive classification entries (IR). Thus, this study infers that the EEG data obtained when the test subjects were attentive possess more identical or similar features, and are easier to recognize. This study also infers that the correct classification ratio of attentiveness is substantially higher than that of inattentiveness, which indicates that the collected attentive EEG data has more similar features and are more easily recognized. However, this does not indicate that inattentive EEG data are not easily recognized, but rather that many causes of inattentiveness exist. Therefore, a greater number of states are hidden within inattentiveness EEG data, necessitating further analysis for identification (e.g., the test subjects were required to multitask by listening to the content of the English sentences, listening to the additional conversations, and considering the content of these conversations). The IR values were not substantial at approximately 50%, which indicates that the system had a 50% probability of failing to detect students' inattentiveness. However, misdetection of students' attentiveness is less likely to affect their emotions during learning. A low AR results in the misjudgment that students are inattentive. If the system signals a warning, students may perceive interference with their studies. Furthermore, 2,400 test samples each were used to calculate AR and IR. However, the actual ratio of attentive to inattentive samples should be considered to determine the overall accuracy rate. For example, hypothesizing that the students were inattentive for 10% of the time during the experiment, the overall accuracy rate would be AR × 0.9 + IR × 0.1. Thus, when students are frequently inattentive, this study recommends adopting a system with a greater cost parameter for EEG classification. Conversely, a system with a lower cost parameter should be adopted when students are generally attentive.

## 5. Conclusions

In traditional classrooms where teachers teach students face to face, the teacher can determine whether students are learning attentively based on their expressions and movements. However, for multimedia or distance learning, teachers experience greater difficulty determining whether students are learning attentively. Therefore, this study employed quantified EEG data to analyze students' learning status, thereby enabling teachers, students, and related personnel to understand whether students are attentive using scientific means. This allows teachers to adjust the teaching content, cultivate students' learning attitudes, and remind students to remain attentive. During the experiment, this study eliminated the features individually. The results showed that the optimum classification accuracy is achieved when five features are used simultaneously. However, the influence that each feature has on the classification accuracy differs. Of the features, the delta value causes the most significant changes, and can influence the classification accuracy by up to 6%. This has been rarely observed in previous studies. According to Penolazzi *et al.* [25], changes in delta activity are related to linguistic acquisition. Therefore, if the learning method belongs to linguistic instruction, the delta feature value would be an important basis for classification. In addition, a previous study has attested that theta and beta activity are not related to attentiveness; however, this study employed more accurate sensing equipment and a greater number of locations when detecting EEG signals. Because of the limitations of a dry sensor for the detection location Fp1, only by using all wavebands can superior classification results be obtained.

The study results indicate that the state of attentiveness is a continuous phenomenon, and that the observation of data from a limited period is slightly superior to the classification accuracy of a single data entry. Furthermore, the EEG signals of attentiveness are easier to identify compared to those of inattentiveness; this is because the EEG signals of inattentiveness contain additional information. However, more unknown data or types of states may be extracted from the EEG signals of inattentiveness. The results also show that using polykernel kernel functions provides greater accuracy compared to using normalized polykernel kernel functions, which indicates that normalizing causes certain figures with a classification value to be obscured, resulting in a poorer classification performance. Currently, when using EEG signals without interference processing for classification, the accuracy rate for successfully detecting that the subject is inattentive is only 50%. EEG interference will be further examined in the future to enhance classification accuracy.

We hope that an immediate recognition system can be developed after EEG data is collected from additional test subjects. This would allow actual application in students' learning environments. Subjects of differing age groups and genders may produce varying EEG signals, which affect the classifier training effectiveness. Therefore, large-scale access of attentive and inattentive EEG signals is essential for future research. Numerous improvements can be made to the study's experimental collection of EEG signal data from subjects using English tests. This study anticipates developing additional methods for identifying subjects' attentiveness in the future. We hope that the methods mentioned in this study, for example, the *k*th NN classifier, artificial neural network, decision tree, and random forest, can be used to assess whether various classification methods improve the recognition accuracy rate of attentive EEG signals.

## Figures and Tables

**Table 1.** The inference of classification accuracy using various features.

		***α*+*β*+*δ*+*θ*+*R***	***β*+*δ*+*θ*+*R***	***α*+*δ*+*θ*+*R***	***α*+*β*+*θ*+*R***	***α*+*β*+*δ*+*R***	***α*+*β*+*δ*+*θ***
Average accuracy for training	PK	76.23	73.13	74.03	68.49	73.61	67.94

	NPK	72.39	69.22	69.74	68.75	69.67	64.12

Average accuracy for *k*-fold	PK	75.87	72.36	74.16	68.63	73.45	67.25

	NPK	71.28	68.96	68.89	67.15	69.18	63.62

Highest accuracy for training	PK	77.96	74.14	75.25	69.43	74.81	68.02

	NPK	73.76	69.97	70.91	69.24	70.35	64.84

Highest accuracy for *k*-fold	PK	76.82	73.95	74.92	69.43	74.34	68.98

	NPK	73.83	70.42	69.73	68.38	70.12	64.46

PK: polykernel, NPK: normalized polykernel.

**Table 2.** AR and IR when employing the polykernel function.

**Cost Parameter**	**Learning State**	**Training**	**k-Fold**
1	AR (Attention)	90.43%	90.64%
	IR (Inattention)	54.26%	55.12%
10	AR (Attention)	90.48%	89.25%
	IR (Inattention)	57.67%	58.04%
50	AR (Attention)	87.23%	86.87%
	IR (Inattention)	59.84%	59.69%
